# Pigs and Men

**Published:** 1858-01

**Authors:** 


### PIGS AND MEN.

WHILE so much is heard of the Hog Cholera, so fatal to that animal in Ohio, said to originate in the swill on which they are fed being so strongly impregnated with strychnine, in order to make a more marketable kind of whisky, the Ohio Legislature contemplates framing a law, visiting with very severe penalties those who thus kill their darling hogs; but as long as men only perished by drinking bad whisky, such a law was not considered specially needed.

The best whisky in the world was made in the secluded locality where we were "born and raised," because it was made of corn or rye only; and we remember many persons who drank *Old Bourbon* daily, and survived their three score years and ten. But it is comparatively a rare thing, now, to see a regular liquor-drinker beyond fifty years of age. Chemists know the reason to be, that liquors are so drugged with poisonous medicines, in order to save time and material, and give them taste, that the intestines are rapidly eaten away, and the most iron constitution is able to resist their destructive agencies but a few short years. Another fact, not less suggestive, not only to Ohio law-makers, but to all others, is, that of six thousand persons, tried last year before the New York Court of Sessions, not more than ninety-four were sober when arrested!

